# Modeling and optimization of bakery production scheduling to minimize makespan and oven idle time

**DOI:** 10.1038/s41598-022-26866-9

**Published:** 2023-01-05

**Authors:** Majharulislam Babor, Olivier Paquet-Durand, Reinhard Kohlus, Bernd Hitzmann

**Affiliations:** 1 Institute of Food Science and Biotechnology, Department of Process Analytics and Cereal Science, University of Hohenheim, 70599 Stuttgart, Germany; 2 Institute of Food Science and Biotechnology, Department of Process Engineering and Food Powders, University of Hohenheim, 70599 Stuttgart, Germany

**Keywords:** Engineering, Mathematics and computing

## Abstract

Makespan dominates the manufacturing expenses in bakery production. The high energy consumption of ovens also has a substantial impact, which bakers may overlook. Bakers leave ovens running until the final product is baked, allowing them to consume energy even when not in use. It results in energy waste, increased manufacturing costs, and CO_2_ emissions. This paper investigates three manufacturing lines from small and medium-sized bakeries to find optimum makespan and ovens’ idle time (OIDT). A hybrid no-wait flow shop scheduling model considering the constraints that are most common in bakeries is proposed. To find optimal solutions, non-dominated sorting genetic algorithm (NSGA-II), strength Pareto evolutionary algorithm (SPEA2), generalized differential evolution (GDE3), improved multi-objective particle swarm optimization (OMOPSO), and speed-constrained multi-objective particle swarm optimization (SMPSO) were used. The experimental results show that the shortest makespan does not always imply the lowest OIDT. Even the optimized solutions have up to 231 min of excess OIDT, while the makespan is the shortest. Pareto solutions provide promising trade-offs between makespan and OIDT, with the best-case scenario reducing OIDT by 1348 min while increasing makespan only by 61 min from the minimum possible makespan. NSGA-II outperforms all other algorithms in obtaining a high number of good-quality solutions and a small number of poor-quality solutions, followed by SPEA2 and GDE3. In contrast, OMOPSO and SMPSO deliver the worst solutions, which become pronounced as the problem complexity grows.

## Introduction

Bakery is one of the major food manufacturing sectors, with steady increases in market share and per capita consumption. Craft bakery sales in Germany in 2021 were 14.9 billion Euros (exclusive of VAT), with an increase of 0.18 billion Euros per year. To meet market demand, the amount of flour consumed, the variety of products developed, and the number of personnel employed have all expanded over the past decade. According to reports, each bakery uses on average of 372 MWh of energy annually, resulting in 101 tons of CO_2_ emissions^1,2^. As the business environment has become more competitive, the objectives for improving the efficiency of a manufacturing system have widened. In order to satisfy customers, meet market demand, and turn a profit, an optimum cost-time profile is crucial. It includes cost savings via the efficient use of assets and materials. Makespan, tardiness, earliness, and energy consumption are some of the most commonly employed cost-cutting objectives in various production environments. However, bakery manufacturing, particularly in small and medium-sized bakeries, is prone to inefficiencies because employees perform many tasks manually for operations that cannot be automated. Furthermore, employee salaries are said to account for a significant amount of the cost^1^. As a result, when planning the production schedule, bakers focus primarily on lowering the makespan.

Production scheduling with more than two machines is a non-deterministic polynomial-time (NP)-hard problem^3,4^. The difficulty of finding the best schedule increases as the number of products, processing stages, and alternative machines for each stage grows. Therefore, the flow shop scheduling problem has been extensively studied to improve the efficiency of several production and service environments, such as bakery^5–7^, glass^8^, steel^9^, wood^10^, chemical process^11^, energy system^12^, healthcare system^13–15^. To put it simply, it is the process of allocating tasks of varying durations from $n$ products to $m$ machines. It also provides supplementary information for assessing a schedule, such as makespan and energy use, that change based on how the tasks are allocated. The most common type of flow shop scheduling problem is the permutation flow shop, in which each product must pass through all the $m$ machines independently in the same order^4^. In complex cases, a processing task of a product may depend on another product. It is known as “no-wait” flow shop scheduling problem when there is no delay allowed between two successive tasks of a product. Many hybrid flow shop models have been developed to reflect the reality, which is mostly specific to a production system^16^. To make production systems energy-efficient and environmentally friendly, many flow shop models have been proposed, which are widely known as “green flow shop model”. Here, in order to establish an efficient resource allocation, total energy consumption is taken into account in addition to makespan^17^.

Although modern industries have been applying many powerful decision-making tools, such as intelligent manufacturing systems, to address complex challenges^18,19^, small and medium-sized bakeries continue to rely on personal experience^6,7^. Furthermore, bakeries' product range and amount change frequently due to market demand and seasonality, demanding continuous monitoring of production efficiency. However, only a few studies have focused on improving bakery manufacturing. In a recent study, Huber and Stuckenschmidt^20^ implemented machine learning approaches to predict hourly sales of bakery items in a retailer store and optimize the baking schedule so that bakers serve customers with fresh products. Nonetheless, the production of a vast number of products, from flour to finished or unfinished goods before delivery to retailers, is a separate segment. In a case study with a medium-sized German bakery, Hecker et al.^6^ observed that the makespan of an existing manufacturing line with 40 products can be lowered by 8.6%. Swangnop et al.^7^ developed a scheduling model for a bakery in Thailand and demonstrated that the existing production, planned based on experience, is inefficient. An additional factor that most bakers overlook is the ovens' energy consumption, which has a vital influence on manufacturing costs and CO_2_ emissions. It has been reported that only baking consumes up to 78% of the total energy depending on the product category^21^. Bakeries typically feature multiple ovens with varied functionalities that are employed according to product specifications^5,6^. As a result, by minimizing the idle time of the ovens, a large quantity of energy can be saved, lowering manufacturing costs and CO_2_ emissions. Babor et al.^5^ investigated a small Spanish bakery and observed that actual production is poorly optimized. The authors weighed the machines' idle time and makespan to the objective function.

The motivation for this study is twofold. First, to solve production optimization problems for bakeries with two objectives: minimizing makespan and energy waste due to oven idle time. It is a hybrid no-wait flow shop scheduling problem because of the following exceptions in bakery manufacturing. Many tasks are carried out manually, and there are numerous substitute machines that can carry out the remaining tasks. Additionally, there are production constraints for a variety of products recipes. Therefore, a mixed-integer linear programming approach for hybrid no-wait flow shop scheduling model (HNFSM) is proposed to simulate bakery production scheduling. The Pareto optimal solutions obtained by multi-objective optimization algorithms are used to analyze the trade-offs between the objectives. Secondly, to compare the performance of five multi-objective optimization algorithms to solve the instances. Because production optimization is time-consuming and performed frequently, attaining optimal solutions in the lowest computation time is essential. We used multi-objective optimization algorithms of two types: evolutionary algorithms and particle swarm optimization-based metaheuristics. Non-dominated sorting genetic algorithm (NSGA-II), strength Pareto evolutionary algorithm (SPEA2), and generalized differential evolution (GDE3) are taken from the former category, while improved multi-objective particle swarm optimization (OMOPSO), and speed-constrained multi-objective particle swarm optimization (SMPSO) are from the latter. To assess their effectiveness, four quality indicators are used: cardinality, convergence, distribution and spread, and convergence and distribution of the obtained solutions. To cluster the solutions into distinct qualities, a Gaussian mixture model^22^ is used.

The following are the contributions of the current study. State-of-the-art multi-objective optimization methods are used to optimize the production efficiency of small and medium-sized bakeries employing a hybrid no-wait flow shop model. By combining various performance metrics, the Gaussian mixture model is used to assess how effectively algorithms solve problems of three complexity levels while varying the number of products and predecessor constraints.

The remainder of the paper is structured as follows. An introduction to bakery production is given in the next section. “Materials and methods” section describes mathematical formulation of a hybrid no-wait flow shop scheduling model to simulate bakery manufacturing. Besides, multi-objective optimization algorithms and their performance indicators are presented. In “Results and discussion” section, the effectiveness of algorithms in solving scheduling problems for bakeries is analysed. “Conclusions and future works” section of this paper provides a summary of findings.

## Bakery production

**Figure 1:**
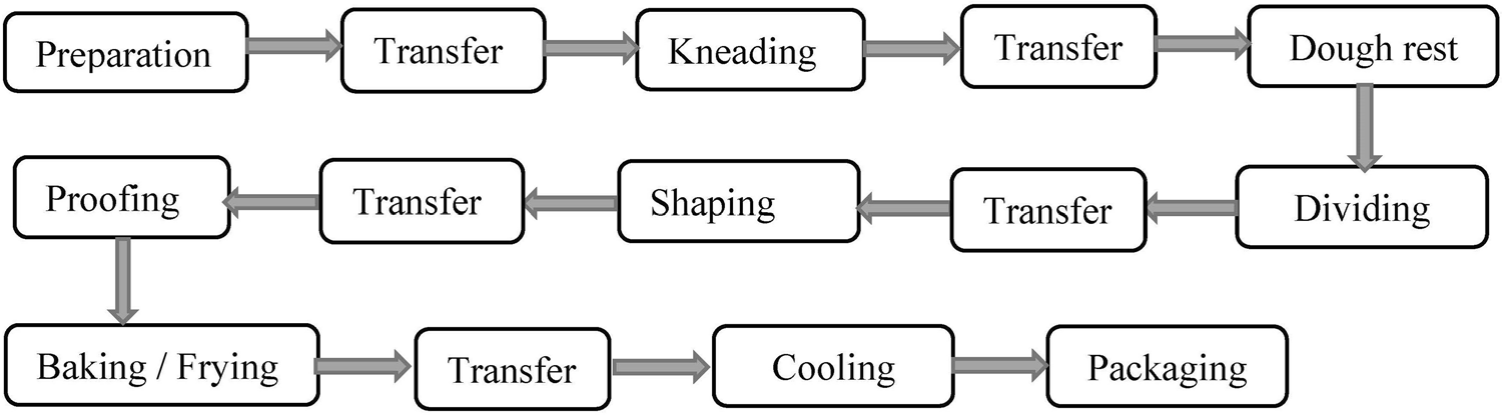
A simplified processing route for bakery products.

A bakery product undergoes a series of processing steps. Each product has a recipe that specifies the order, duration, and machines that will be utilized to complete the tasks. Figure 1 shows a simplified processing route. Making the dough starts by mixing and kneading the ingredients, such as flour, water, and salt. In most cases, yeast is added to induce fermentation, which produces the leavening agent CO_2_ and other aroma precursors. If small and medium-sized bakeries are considered, the transfer of unfinished products from one machine to another is performed manually by employees almost after every processing task. The fermentation process has a vital impact on the final product’s quality. Because temperature and humidity have a considerable impact on yeast sugar fermentation, the duration of the processing stages under various conditions is strictly controlled. Performing one task longer than the predefined duration results in overtreatment and consequently, delay for the following tasks, and a loss of product quality, both of which are undesirable. Therefore, as soon as ingredients are mixed and kneaded, the next stages are carried out with no delay.

To avoid wasting energy, bakers turn off machines during the idle time—the time between two scheduled operations. However, ovens need preparation time to reach the set temperature before performing baking. Turning them off after an operation requires a restart well ahead of the next operation. When they are turned off, no energy is consumed, but the temperature steadily declines. If the idle time between two tasks is short, the temperature drops less, and the time required to re-heat is reduced. However, if the temperature drops sharply, such as due to chilly weather, the assumed time may not be long enough to reach the set point. Again, with the prolonged idle period, the right time to restart the ovens must be considered to avoid wasting energy and have them ready at the proper time. When the number of products is large and there are many manual tasks to perform, it is difficult for bakers to keep track. The following tasks must be postponed accordingly if the oven's temperature is not up to the set point in time. It may affect the product quality and lead to inefficient production. To avoid these consequences, bakers keep the ovens running throughout the production time.

The duration and machine set up for processing steps are predetermined. Hence, the energy consumption during operational time is constant regardless of how optimized a schedule is. In contrast, the effectiveness of the schedule influences oven idle time, which has a direct impact on the quantity of wasted energy. Moreover, small, and medium-sized manufacturers have limitations in recording energy data for each device. In this case, the idle time of the ovens can be an ideal indicator of energy waste. Machine idle time has been investigated in various studies as one of the objectives for optimizing production schedule^5,6,23,24^.

## Materials and methods

In this paper, three bakery production optimization problems, labelled with BK15, Bk40 and BK50 were solved. The number in labels specifies how many products were produced in each manufacturing line, for example, the dataset BK15 contains production information for 15 bakery products. BK15 and BK50 were taken from Babor and Hitzmann^25^ and BK40 was taken from Hecker et al.^6^. BK15 had three employees, eight machines, and two ovens with four compartments; BK40 had eleven machines, three ovens, and nine compartments; and BK50 had ten employees, fourteen machines, and three ovens, and ten compartments. In bakeries, many ovens have separate compartments, each of which can be used independently to bake a batch of products. In the following discussion, the problems are labelled according to the approximate number of total products. The implementation and simulation of HNFSM and multi-objective optimization algorithms were performed using the computer language Python (version 3.7)^26^ on a computer running Microsoft Windows 10 as the operating system with a configuration of an Intel Core i5 at 4 × 3.20 GHz, 8 GB ram.

### Problem definition and scheduling model

**Figure 2:**
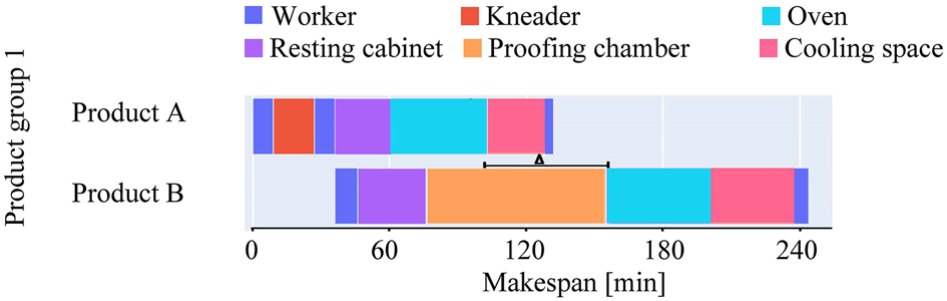
Gantt chart showing schedule of one product group with unified initial stages. The triangle (∆) shows the oven idle time.

In small, and medium-sized bakeries, using the same dough for various products made from the same ingredients is a frequent practice. This practice takes advantage of the machines' capabilities in the initial stages to reduce preparation time. Bakers split the dough after completing a few processing tasks into various parts. It enables the products to be treated differently in subsequent phases to meet recipe requirements. There is no common procedure for separating dough as it completely depends on the type of products and recipes. Figure 2 illustrates a schedule of two products that are produced from the same dough and shared the same processing machines at the initial phase.

**Figure 3:**
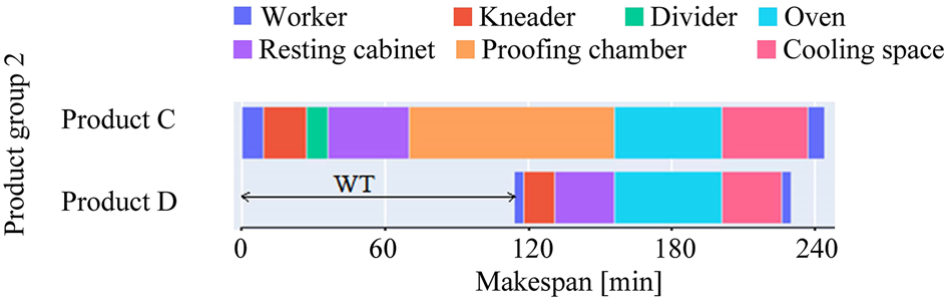
Simplified schedule for one product group where baking is performed together. Here WT is the difference between the start time of Product C and Product D.

In another scenario, multiple products that came across different processing routes are baked in the same oven. Because baking consumes high energy, running an oven while it is only partially occupied causes energy waste. Figure 3 shows a schedule for two products where baking is performed together. In both cases (Figs. 2, 3), the products are internally dependent such that their common tasks must be performed at the same time. This preceding rule is used to arrange products into groups in the flow shop model. Only one product in a group has no predecessor, which means it can be scheduled at any time throughout the production runtime. However, the schedules for the rest of the products in that group depend on it. Table 1 represents simplified production data for one product group which is visualized in Fig. 2.

**Table 1:** Simplified production data for one product group.

Group	Product	Bowl time [min]	Name	Processing stage	Duration [min]	Machine/Employee
1	1	0	Product A	Preparation	9	Employee
				Kneading	18	Kneader
				Dividing	9	Employee
				Dough rest	25	Resting cabinet
				Baking	42	Oven
				Cooling	25	Cooling space
				Packaging	4	Employee
	2	36	Product B	Dividing	10	Employee
				Dough rest	30	Resting cabinet
				Proofing	80	Proofing chamber
				Baking	45	Oven
				Cooling	36	Cooling space
				Packaging	7	Employee

In reality, many product groups are organized based on their internal dependence. Within a group, each product has an individual bowl time. It indicates the start time difference between a predecessor product and any other product in a group. For one processing stage, there might be multiple alternative machines and employees, from which one should be selected based on availability. In general, for dough rest and cooling, no energy-consuming machines are required and therefore are considered to have the capacity to operate as many products as possible at a time. Similarly, due to having enough space in proofing chambers, it is assumed that the proofing stage has no blockage. Considering the bakers’ practice, ovens can operate multiple products from the same product group at a time (Fig. 3). The rest of the machines can perform a task only from one product.

**Figure 4:**
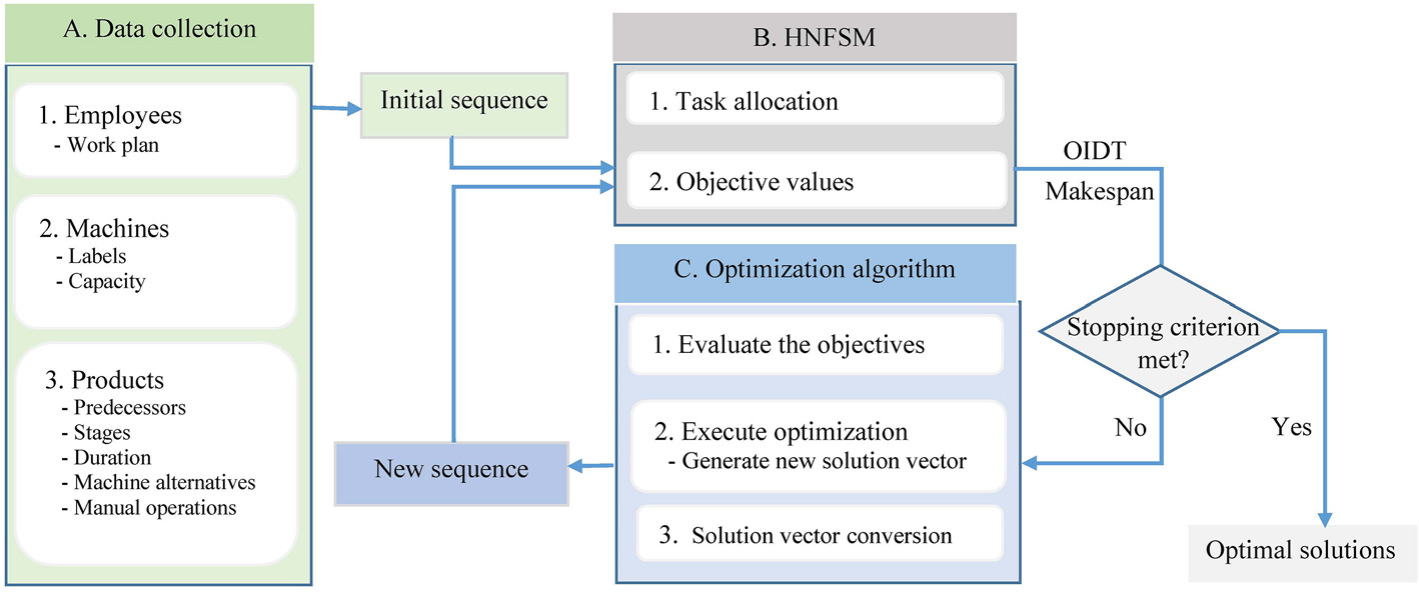
Schematic diagram of bakery production optimization using hybrid no-wait flow shop scheduling model (HNFSM).

Figure 4 shows the procedure of optimizing the bakery production schedule. It can be discussed in three distinct segments: data collection, HNFSM, and optimization algorithm. Information about bakery products, machines, and employees is recorded during data collection. Depending on the internal dependence the products are sorted into distinct groups. An initial product sequence, an order of product groups in which they are produced, is transferred to HNFSM. In HNFSM, the processing tasks are allocated among the machines and employees. Here, the actual scheduling, i.e., exact start and end time, machine, or employee to conduct a task is determined. Like other flow shop models, the products that are placed first in the order will get priority in occupying the machines and employees. The following products are scheduled according to machine availability. The makespan and OIDT are calculated as a quality indicator for a schedule, which is considered as a baseline to start improving. The optimization algorithm proposes a new candidate solution vector, which requires to convert into a product group sequence to use for HNFSM. The candidate solution conversion approach is explained later. This procedure is repeated until a certain termination criterion is met.

This section describes the mathematical formulation of the proposed hybrid no-wait flow shop scheduling model. Table 2 defines the notions that are used to describe HNFSM. Ovens at bakeries typically contain multiple compartments that may each be used separately to bake different products. OIDT may exceed the makespan since it calculates the total idle time of all oven compartments. The following equations are used to calculate makespan and OIDT.

M1 $$ Makespan = max\left( {CT_{g,p,s} } \right)\quad \forall g \in PG, \forall p \in P $$

M2 $$ OIDT = \sum\nolimits_{k = 1}^{m} {\left( {end_{k} - start_{k} - \sum\nolimits_{g = 1}^{NG} {\sum\nolimits_{p = 1}^{n} {PT_{g,p,k} } } } \right)\quad \forall g \in PG, \forall p \in P, \forall k \in V} $$

**Table 2:** Notations used in hybrid no-wait flow shop scheduling model (HNFSM).

Notation	Description
$NG$	Number of groups of products
$n$	Number of products in a group
$m$	Number of machines
$e$	Number of employees
$g$	Index of groups;$g = 1, 2, \dots , NG$
$p$	Index of products in group g; $p = 1, 2, \dots , n$
$k$	Index of machines;$k = 1, 2, \dots , m$
$l$	Index of employees;$l=1, 2, \dots , e$
$s$	Index of the processing stage
$t$	Index of production runtime in minutes
$PG$	Set of product groups;$G = \{1, 2, \dots , NG\}$
$P$	Set of products in a group $g$; $P = \{1, 2, \dots , n\}$
$M$	Set of machines;$M = \{1, 2, \dots , m\}$
$V$	Set of oven compartments;$V\subset M$
$U$	Set of machines with unlimited capacity;$U\subset M$
$E$	Set of employees;$E = \{1, 2, \dots , e\}$
${WT}_{g, p}$	Time difference between product $p$ and its predecessor in group $g$
${PT}_{g,p,s}$	Processing time at stage $s$ of product $p$ in group $g$
${PT}_{g,p,k}$	Processing time of product $p$ in group $g$ processed by machine $k$
${ST}_{g,p,s}$	Start time for the operation at stage $s$ of product $p$ in group $g$
${CT}_{g,p,s}$	Completion time of stage $s$ of product $p$ in group $g$
${start}_{k}$	The time when machine $k$ starts its first operation
${end}_{k}$	The time when machine $k$ finishes its last operation
${start}_{l}$	The time when employee $l$ starts the work
${end}_{l}$	The time when employee $l$ finishes the work
${O}_{g, p,s,k}$	$$\left\{ {\begin{array}{*{20}l} {1, } \hfill & {{\text{if }}\,{\text{the }}\,{\text{product}}\,p\,{\text{in }}\,{\text{group}}\,g\,{\text{is }}\,{\text{processed }}\,{\text{on }}\,{\text{machine}}\,k\,{\text{at }}\,{\text{stage}}\,s} \hfill \\ {0,} \hfill & {{\text{if}}\,{\text{ otherwise}}} \hfill \\ \end{array} } \right.$$
$O_{g, p,s,l}$	$$\left\{ {\begin{array}{*{20}l} {1, } \hfill & {{\text{if }}\,{\text{the }}\,{\text{product}}\,p\,{\text{in }}\,{\text{group}}\,g\,{\text{is }}\,{\text{ processed}}\,{\text{ by }}\,{\text{machine}}\,l\,{\text{at }}\,{\text{stage}}\,s} \hfill \\ {0,} \hfill & {{\text{if }}\,{\text{otherwise}}} \hfill \\ \end{array} } \right.$$

The HNFSM is described as follows.

M3 $$ Min \left( {Makespan} \right) $$

M4 $$ Min \left( {OIDT} \right) $$

Subject to

M5 $$ ST_{g,1,1} \ge 0\quad \forall g \in PG $$

M6 $$ ST_{g,p,1} = ST_{g,1,1} + WT_{g, p } \quad \forall g \in PG, \forall p \in P \backslash \left\{ 1 \right\} $$

M7 $$ PT_{g, p,s} > 0\quad \forall g \in PG, \forall p \in P, \forall k \in M $$

M8 $$ CT_{g,p,s} = ST_{g,p,s} + PT_{g,p,s} \quad \forall g \in PG, \forall p \in P $$

M9 $$ ST_{g,p,s + 1} = CT_{g,p,s} \quad \forall g \in PG,\forall p \in P $$

M10 $$ \sum\nolimits_{k = 1}^{m} {O_{g, p,s,k} \le 1} \quad \forall g \in PG, \forall p \in P, \forall k \in M $$

M11 $$ \sum\nolimits_{g = 1}^{NG} {\sum\nolimits_{p = 1}^{n} {\sum\nolimits_{{t = ST_{g,p,s} }}^{{CT_{g,p,s} }} { O_{g,p,s,k} \le \left( {CT_{g,p,s} - ST_{g,p,s} } \right)} } } \quad \forall g \in PG, \forall p \in P, \forall k \in M \backslash \left( {U \cup V} \right) $$

M12 $$ \sum\nolimits_{g = 1}^{NG} {\sum\nolimits_{p = 1}^{n} {\sum\nolimits_{{t = ST_{g,p,s} }}^{{CT_{g,p,s} }} {O_{g,p,s,k} \le n\left( {CT_{g,p,s} - ST_{g,p,s} } \right) } } } \quad \forall g \in PG, \forall p \in P, \forall k \in V $$

M13 $$ \sum\nolimits_{g = 1}^{NG} {\sum\nolimits_{p = 1}^{n} {\sum\nolimits_{{t = ST_{g,p,s} }}^{{CT_{g,p,s} }} {O_{g,p,s,k} \le \sum\nolimits_{g = 1}^{N} n } } } \quad \forall g \in PG, \forall p \in P, \forall k \in U $$

M14 $$ start_{l} < end_{l} \quad \forall l \in E $$

M15 $$ \mathop \sum \limits_{l = 1}^{e} O_{g, p,s,l} \le 1\quad \forall g \in PG, \forall p \in P, \forall l \in E $$

M16 $$ \sum\nolimits_{g = 1}^{NG} {\sum\nolimits_{p = 1}^{n} {\sum\nolimits_{{t = ST_{g,p,s} }}^{{CT_{g,p,s} }} { O_{g,p,s,l} \le \left( {CT_{g,p,s} - ST_{g,p,s} } \right)} } } \quad \forall g \in PG, \forall p \in P, \forall l \in E $$

M17 $$ ST_{g,p,s} \ge start_{l} \quad \forall O_{g, p,s,l} = 1, \forall g \in PG, \forall p \in P, \forall l \in E $$

M18 $$ CT_{g,p,s} \le end_{l} \quad \forall O_{g, p,s,l} = 1, \forall g \in PG, \forall p \in P, \forall l \in E $$

M19 $$ \sum\nolimits_{k = 1}^{m} {O_{g, p,s,k} } + \sum\nolimits_{l = 1}^{e} {O_{g, p,s,l} } \le 1\quad \forall g \in PG, \forall p \in P, \forall k \in M, \forall l \in E $$

The objective functions are shown in Eqs. (M3), (M4). Constraint (M5) states that the start time for the predecessor product of any group can be $\ge 0$. Constraint (M6) defines it for successor products in the group. It includes a time difference between the start time of predecessor and successor products. Constraint (M7) declares that the processing time for any stage must be greater than 0 min. The no-wait condition between two consecutive stages of a product is defined by conditions (M8) and (M9). Constraint (M10) ensures that an operation from a product can occupy only one machine. A machine can only perform one task at a time except for the ovens $(k\in V)$ and the machines with unlimited capacity $(k\in U)$, as defined by constraint (M11). Constraint (M12) allows ovens to bake multiple products from the same group. According to constraint (M13), machines with unlimited capacity can perform any number of tasks at a time. Condition (M14) validates the shift plan of employees. Constraint (M15) limits the number of employees assigned to a single task. Constraint (M16) restricts the number of tasks assigned to a single employee at any given time. Employee job allocation is limited by constraints (M17) and (M18) to be within their working hours. Condition (M19) states that either an employee or a machine limitation can be occupied for a task. However, in the bakery process, some tasks require no machine and employee, such as dough rest.

### Multi-objective optimization

Most real-world optimization problems that scientists and engineers handle routinely are multi-objective problems, where systems demand satisfying more than one parameter. Conventionally, such problems are simplified in two diverse ways: after converting multiple objectives into one by using the linear weighting method and featuring objectives as constraints. These approaches provide an optimized solution to a satisfactory level without handling the complex interrelations between multi-objectives. Nonetheless, depending on the type of problems, these approaches have limitations. The former method relies on personal preference when determining the importance of objectives, which has a major impact on the solution. Furthermore, weighting factors might lead the optimizer to a poor solution when solving a problem with a non-convex Pareto front that is unknown beforehand. The latter approach struggles to deal with the high-dimensional, multi-objective optimization problems and is prone to producing suboptimal solutions. In reality, many multi-objective optimization problems do not show continuous solutions in the objective space. Therefore, if objectives are restricted to different ranges, for an optimizer it is challenging to find an optimal solution that meets these constraints. There are many optimal solutions to multi-objective problems with many local minima in a multi-objective space. Following traditional methods, the entire procedure must be repeated many times, each time adjusting the weighting factors or constraints for the objectives to make sure that the obtained solution is not one of these local minima. However, there is no guarantee that a complete set of optimum solutions will be obtained. To address this problem, several multi-objective optimization algorithms have been proposed^27–33^. Given a decision space $\chi$ mapped into ${\mathbb{R}}$ for $q$ objective functions ${f}_{1}: \chi \to {\mathbb{R}},\dots ,{f}_{q}: \chi \to {\mathbb{R}}$, a multi-objective optimization minimization problem can be stated as follows (Eq. 1).

1 $$ min f_{1} \left( x \right), \ldots , min f_{q} \left( x \right); x \in \chi \,{\text{and}}\,q > 1 $$

where ${f}_{1}(x), \dots , { f}_{q}(x)$ are objective functions such that minimizing one function leads to an increase in others.

**Figure 5:**
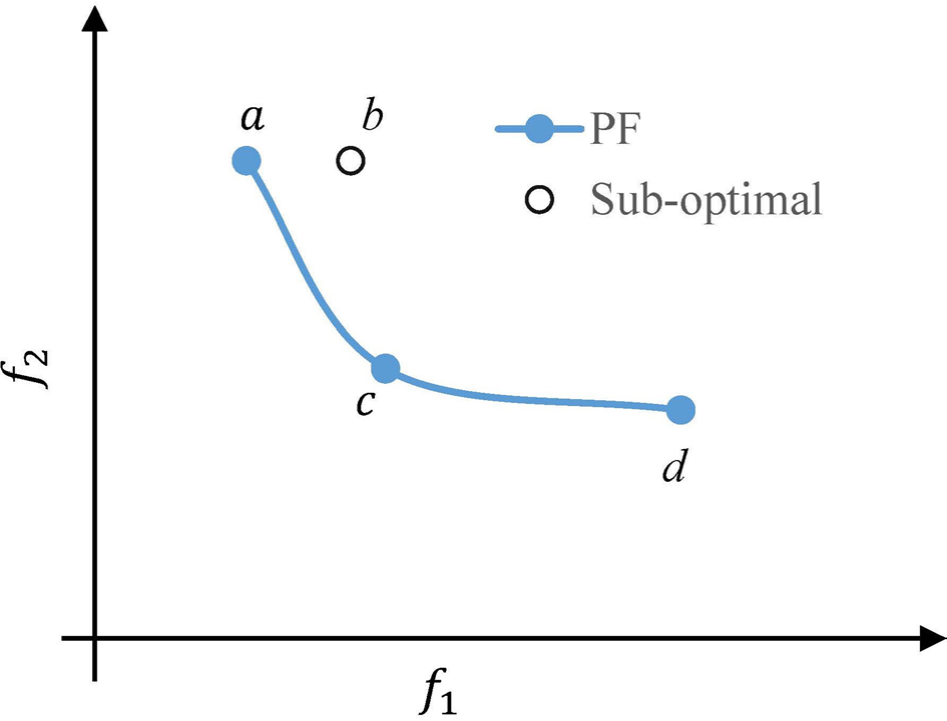
Concept of Pareto dominance for bi-objective functions (${f}_{1}$ and ${f}_{2}$) optimization minimization problem.

Multi-objective optimization, unlike single-objective optimization, generates a collection of optimal solutions by displaying tradeoffs between the objectives in the objective space^24^. Therefore, an objective vector has $q$ values, such as ${[ f}_{1}(x), ..., {f}_{q}(x)]$, each of which reflects the extent of the corresponding objective. Figure 5 outlines an objective space for a bi-objective $(q=2)$ problem. The multiple optimal solutions in this space are selected such that they show the best tradeoffs between the objectives, which is defined by Pareto dominance. Pareto dominance is the fundamental of multi-objective optimization algorithms, extensively used to distinguish optimal solutions from suboptimal solutions. To define the Pareto dominance, given two objective vectors, $\overrightarrow{\mathrm{a}} =[{a}_{1}, ..., {a}_{q}]$ and $\overrightarrow{\mathrm{b}} =[{b}_{1}, ..., {b}_{q}]$ and $\overrightarrow{\mathrm{a}}$ is said to dominate $\overrightarrow{\mathrm{b}}$($\overrightarrow{\mathrm{a}}\preccurlyeq \overrightarrow{\mathrm{b}}$) if and only if ${\overrightarrow{\mathrm{a}} }_{\mathrm{d}}\le {\overrightarrow{\mathrm{b}} }_{\mathrm{d}}$ for every ${\text{d}} \in \left\{ {1, \ldots , {\text{q}}} \right\}$ and ${\overrightarrow{\mathrm{a}} }_{\mathrm{d}}<{\overrightarrow{\mathrm{b}} }_{\mathrm{d}}$ for at least one of ${\text{d}} \in \left\{ {1,{ } \ldots ,{\text{ q}}} \right\}$. In words,$\overrightarrow{\mathrm{a}}$ dominates $\overrightarrow{\mathrm{b}}$, if $\overrightarrow{\mathrm{a}}$ is not worse in any objective and better in at least one objective than $\overrightarrow{\mathrm{b}}$^34,35^. Figure 5 shows that the objective vector $\overrightarrow{\mathrm{a}}$ dominates $\overrightarrow{\mathrm{b}}$ as it improves ${f}_{1}$ while not worsening ${f}_{2}$. However, considering $\overrightarrow{\mathrm{a}}$, $\overrightarrow{\mathrm{c}}$ and $\overrightarrow{\mathrm{d}}$, no one dominates none and thus together they form the Pareto front (PF).

### Optimization algorithms

The concept of Pareto dominance has been used fundamentally in these multi-objective optimization algorithms to find a collection of optimal solutions from a population that progresses over the generations. The strength Pareto evolutionary algorithm (SPEA) is one that was later improved to SPEA2 by eliminating a few weaknesses^32^. Similarly, the non-dominated sorting genetic algorithm (NSGA) was improvised for NSGA-II by reducing the computation complexity using a fast non-dominated sorting approach^27^. The inclusion of elitism, a feature that preserves the good solutions over generations, in NSGA-II makes it comparable with SPEA2. The third version of generalized differential evolution (GDE3), which originated from the differential evolution algorithm, is relatively a new member of this group^36^.

Based on the simulation of the social behavior of birds, the particle swarm optimization algorithm was first proposed by Eberhart and Kennedy^37^**.** This concept has been used in several studies to develop multi-objective optimization algorithms^34,34,38–45^. In an improved particle swarm optimization-based algorithm, known as OMOPSO, Pareto dominance, crowding distance, and mutation operators are included, resulting in highly competitive performance^44^. Later, an extended version, speed-constrained multi-objective particle swarm optimization (SMPSO), was introduced. It is reportedly aimed to adapt particle velocity when it gets higher to generate an effective position in the search space^42^.

However, there is no guarantee that all MOA solutions are truly optimal for an unknown problem. In most previous studies, the effectiveness of multi-objective optimization methods has been demonstrated by solving different mathematical test functions. Solving real-world high-dimensional production scheduling problems are computationally expensive and rarely used as benchmarks to test algorithms. A few studies applied these state-of-art multi-objective algorithms to solve scheduling problems^46–51^. In Annex A, the algorithms are briefly described.

### Solution vector conversion

The optimization candidate solution is a vector consisting of continuous values of size equal to the number of product groups in a problem. In contrast, a solution to HNFSM is a set of discrete numbers, where each number represents one product group. The order of these discrete numbers makes the difference in the final schedule as it implies when each product should be produced. Therefore, a conversion of the algorithmic solution is required. To convert the solution vector into a set of discrete numbers, the smallest position value rule is employed. Table 3 explains the candidate solution conversion procedure for 3 product groups using the smallest position value rule. In this approach, the index of each value in the solution vector is conjugated with a product group. The indexes are sorted by the rule of smallest to the largest value in the vector. The sorted index is used as a solution to the problem. The order of the numbers in the product sequence specifies the order of conjugated product groups in which they should be produced.

**Table 3:** Conversion of a sequence vector to a product group sequence using smallest position value rule.

Generation $(G)$	Sequence vector $({\overrightarrow{x}}_{i,G})$	Sorted index	Product group sequence
1	[1.22, − 1.08, 1.90]	[1–3]	{2, 1, 3}
2	[− 0.29, 0.25, 0.80]	[1–3]	{1, 2, 3}
3	[1.06, 0.30, -0.20]	[1–3]	{3, 2, 1}

### Performance indicators

Ye et al.^52,53^ have described the difficulty in achieving effectiveness and efficiency while finding optimized solution to no-wait flow shop scheduling problems. In this study, the performance of an algorithm is evaluated based on obtained Pareto front, called candidate PF, with the true Pareto front (PF*) to a problem. Initially, PF* for a problem is unknown. Once the candidate fronts (PF) for a problem are obtained by algorithms, the PF* is calculated by taking only Pareto optimal solutions from them.

#### Cardinality

To measure the cardinal quality of optimal solutions, Pareto domination strength is used, which considers the number of optimal and non-optimal solutions in a candidate PF obtained by any algorithm. Pareto domination strength was calculated by using Eq. (2). A higher Pareto domination strength indicates the worst performance of an algorithm.

2 $$ PDS = { }\frac{{\left| {\left\{ {a:{ }a{ } \in { }PF\,{\text{ and}}\,{ }a{ } \notin { }PF^{*} } \right\}} \right|{ } - { }\left| {\left\{ {a:{ }a{ } \in { }PF\,{\text{ and }}\,a{ } \in { }PF^{*} } \right\}} \right|{ }}}{{\left| {{ }PF{ }} \right|{ } \times { }\left| {{ }PF^{*} } \right|}} $$

where $PDS$ is Pareto domination strength, $\left|.\right|$ indicates cardinality of a set, *a* is an objective vector.

#### Distribution and spread

The maximum spread of the solutions in a front captures the spread of the solutions in a front using Eq. (3). A higher value for this indicator represents that an algorithm performed better.

3 $$ MSF = \left[ {\frac{1}{Q}{ }\mathop \sum \limits_{q = 1}^{Q} \left[ {\frac{{min\left( {f_{q}^{max} ,{ }F_{q}^{max} } \right){ } - { }max\left( {f_{q}^{min} ,{ }F_{q}^{min} { }} \right)}}{{F_{q}^{max} - { }F_{q}^{min} }}} \right]^{2} } \right]^{1/2} $$

where $MSF$ is maximum spread of the solutions in a front, $Q$ is the number of objectives, ${f}_{q}^{max}$ and ${f}_{q}^{min}$ are the maximum and minimum values of the *qth* objective in PF*, respectively, ${F}_{q}^{max}$ and ${F}_{q}^{min}$ are the maximum and minimum values of the *qth* objective in the *PF* provided by the algorithm that is under evaluation.

#### Convergence

Convergence measures the degree of proximity between PF* and its approximation, e.g., candidate *PF* obtained by an algorithm. As a convergence indicator, distance to the Pareto front represents how close the solutions of two fronts are. A higher distance to the Pareto front, calculated by using Eq. (4), indicates an algorithm performed worst.

4 $$ DPF = \frac{1}{{\left| { PF^{*} } \right|}} \left( {\mathop \sum \limits_{{a \in PF^{*} }} \mathop {\min }\limits_{ b \in PF} Ed\left( {a,b} \right)} \right) $$

where $DPF$ is distance to the Pareto front, $Ed\left(a,b\right)$ is the Euclidean distance between objective vectors $a$ and $b$, PF^*^ is the true Pareto front, and *PF* is the front obtained by an algorithm that is under evaluation.

#### Convergence and distribution

**Figure 6:**
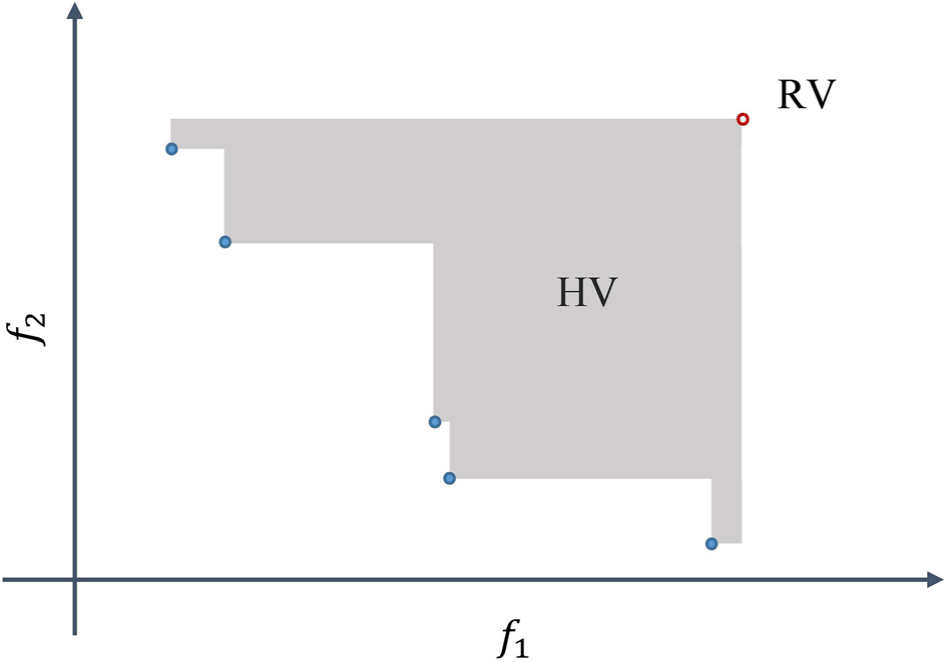
Hypervolume (HV) of one front for two objective functions (${f}_{1}$ and ${f}_{2}$). Filled circles are solutions in front and empty circle is a reference objective vector (RV).

The hypervolume of the front in objective space describes the convergence and distribution of the solutions obtained by an algorithm. It calculates the volume of the space covered by the solutions of a front and delimited from above by a reference objective vector. It defines the upper limit for each objective in the objective space to consider for calculating hypervolume (Fig. 6) by using Eq. (5). A higher relative hyper volume indicates that an algorithm performed better.

5 $$ HP_{PF} = \lambda_{q} \left( {\bigcup\nolimits_{a \in PF } {\left[ {a, RV} \right]} } \right) $$

where ${\lambda }_{q}$ is the *q-*dimensional Lebesgue measure, *PF* is a front obtained by an algorithm that is under test, $a$ is an objective vector and *RV* is a reference objective vector. In this study, for the two objectives, relative hyper area (Eq. 6) is used to compare the performance of different algorithms.

6 $$ RHA \left[ \% \right] = \frac{{HP_{PF} \times 100}}{{HP_{{PF^{*} }} }} $$

where $RHA$ is relative hyper area, *HP*_*PF*_ and *HP*_PF*_ are hyper volumes for a front obtained by an algorithm that is under evaluation and true Pareto front for a problem, respectively.

## Results and discussion

### True Pareto front and candidate solutions

**Figure 7:**
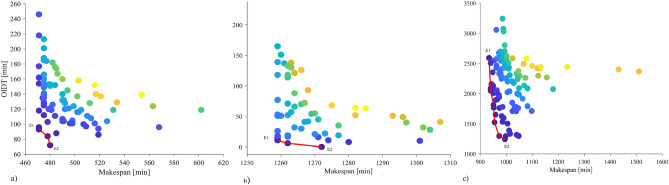
Candidate solutions represented by their quality indicators (circles) obtained by five optimization algorithms and true Pareto front (line) for problem (**a**) BK15, (**b**) BK40 and (**c**) BK50. The color gradient of the circles represents the quality of the solutions, with the darkest blue being the best and pale yellow being the worst.

Figure 7 shows quality indicators representing candidate solutions in objective space obtained by algorithms. True Pareto front (PF*) is the line that connects only a set of optimal solutions. PF* for the instances has only a few solutions, even though many sub-optimal solutions exist nearby. BK15 (Fig. 7a) and BK40 (Fig. 7b) have three Pareto solutions each, but BK50 has seven (Fig. 7c). For BK15 and BK40, the difference between boundary solutions (E1 and E2), which represent extreme tradeoffs between objectives inside PF*, is insignificant. Boundary solutions for BK15 reveal tradeoffs within a 9 min difference in makespan and 21 min difference in OIDT, while they are 13 min and 11 min differences for BK40, respectively. In contrast, due to a 61 min rise in makespan, PF* for BK50 conveys a large 1348 min decline in OIDT. Unlike many mathematical function optimizations described in previous studies^31,36,42^, the solutions in PF* are not continuous in objective space. Furthermore, PF* may have a convex or concave form, which adds to the complexity of solving higher-dimensional problems for optimizers.

Despite a dramatic drop in OIDT over the PF*, BK50 has the highest OIDT (1243 min) at E2. The reason could be that many products in BK50 have predecessors. If multiple products within a group require different specific ovens and the number of products in the group is higher, it is most likely to have higher oven idle time. Candidate solutions that are more densely dispersed in the higher OIDT area support it, with only a few solutions found around E2 (Fig. 7c). In contrast, BK15 has only 3 groups with more than one product and BK40 has no group with multiple products. The PF* of these cases shows a minimum OIDT of 72 min and 0 min, respectively at the E2 point. In addition, their candidate solutions are distributed throughout the objective axis (Fig. 7a,b). BK50 has many groups where the initial stages are processed combinedly to take advantage of machine capacity and save preparation time. Since these products eventually require different baking ovens, finding these ovens available at different time spans leads to a higher OIDT.

**Figure 8:**
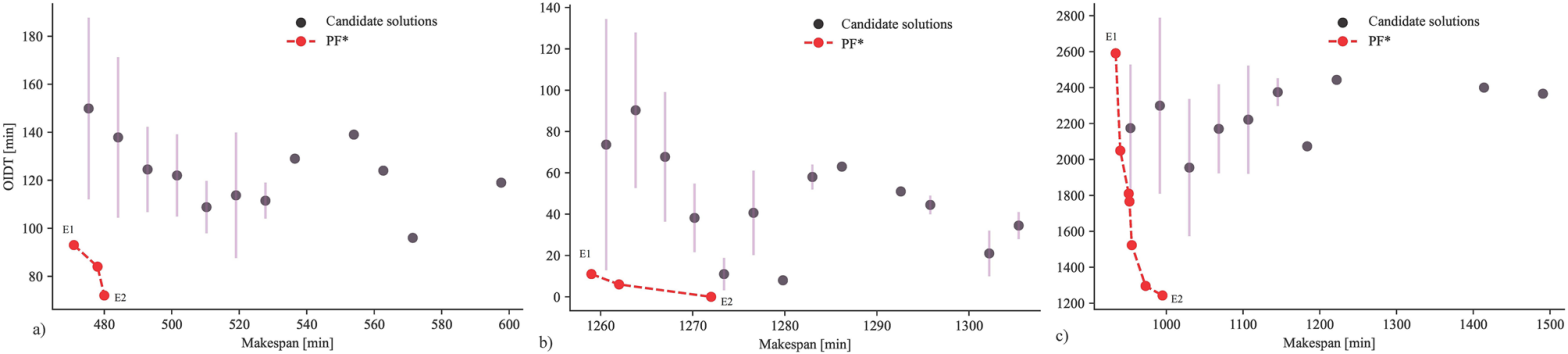
Binned scatter plot of quality indicators representing candidate solutions obtained by five algorithms for instances: (**a**) BK15, (**b**) BK40 and (**c**) BK50. Error bars represent the standard deviation of OIDT.

Multiple solutions were achieved at the shortest makespan (E1) in all cases, but they were dispersed unevenly, with a few having significantly larger OIDT than the respective Pareto solution (E1). The binned scatter plot (Fig. 8) gives two indications. Firstly, a schedule with minimum makespan does not guarantee to have minimum OIDT. A similar result was observed in previous studies^5,23^. Therefore, a schedule optimized with a goal to minimize the makespan might be highly inefficient in energy usage. For example, the candidate solutions for BK15 above E1 shows up to 150 min higher OIDT compared to E1 despite having the same makespan, which is even higher for BK40 (231 min). Figure 8 demonstrates that the error bars are pronounced in the region of the lower makespan. It implies that there is a high possibility an optimizer will produce poor solutions around the shortest makespan with a higher energy waste due to OIDT.

Secondly, solutions with a marginal increase in the shortest makespan could result in an acute reduction in OIDT. As a result, a substantial amount of energy can be saved, lowering operational expenses and CO_2_ emissions. Because the makespan dominates manufacturing cost, the gain in OIDT is compared to the loss in makespan from the shortest makespan at E1. If any Pareto solution other than E1 offers an intense reduction in OIDT while losing a marginal amount of makespan, the entire manufacturing cost can be reduced even more. For example, for BK50, E2 offers OIDT drop by 8% for each percentage increase in makespan from E1 (Fig. 8c). In other words, E2 is more efficient than E1 since it lowers OIDT by 1348 min while increasing makespan by only 61 min.

### Candidate Pareto front

**Figure 9:**
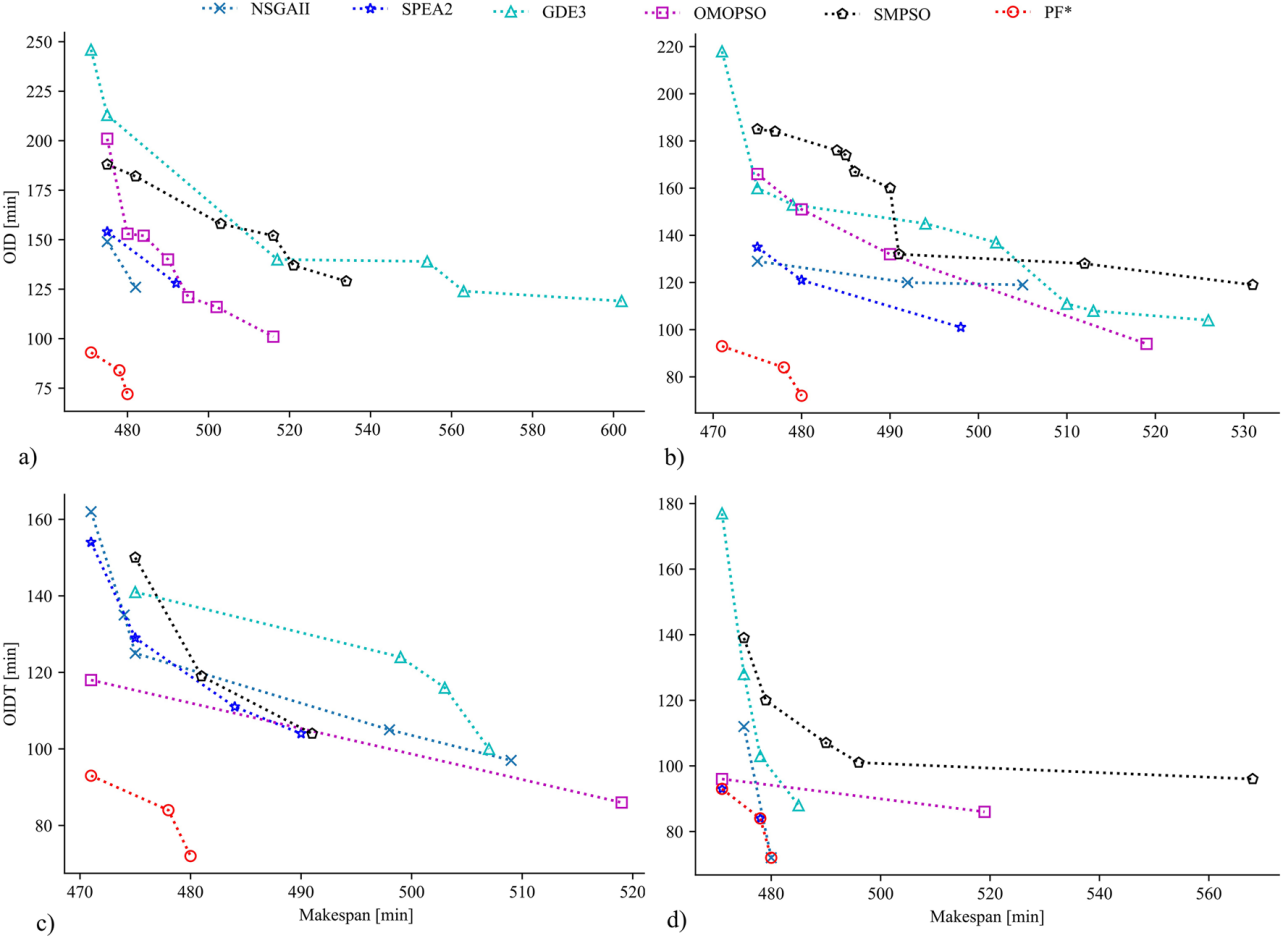
Candidate Pareto front (PF) for BK15 from optimization algorithms with—(**a**) 50 iterations, (**b**) 100 iterations, (**c**) 200 iterations and (**d**) 300 iterations.

Figure 9 shows the candidate PF for BK15 attained by algorithms. For 50 and 100 iterations, NSGA-II and SPEA2 showed better performance. However, with increasing the iteration size, OMOPSO and GDE3 obtained improved solutions too. In contrast, despite offering a high number of solutions, SMPSO displayed comparatively poor performance. A similar performance was observed for BK50 (not shown). In contrast, for BK40 (not shown), the NSGA-II performed worst compared to OMOPSO and SMPSO. SPEA2 always found only one solution, though it was close to being the optimal solution.

**Figure 10:**
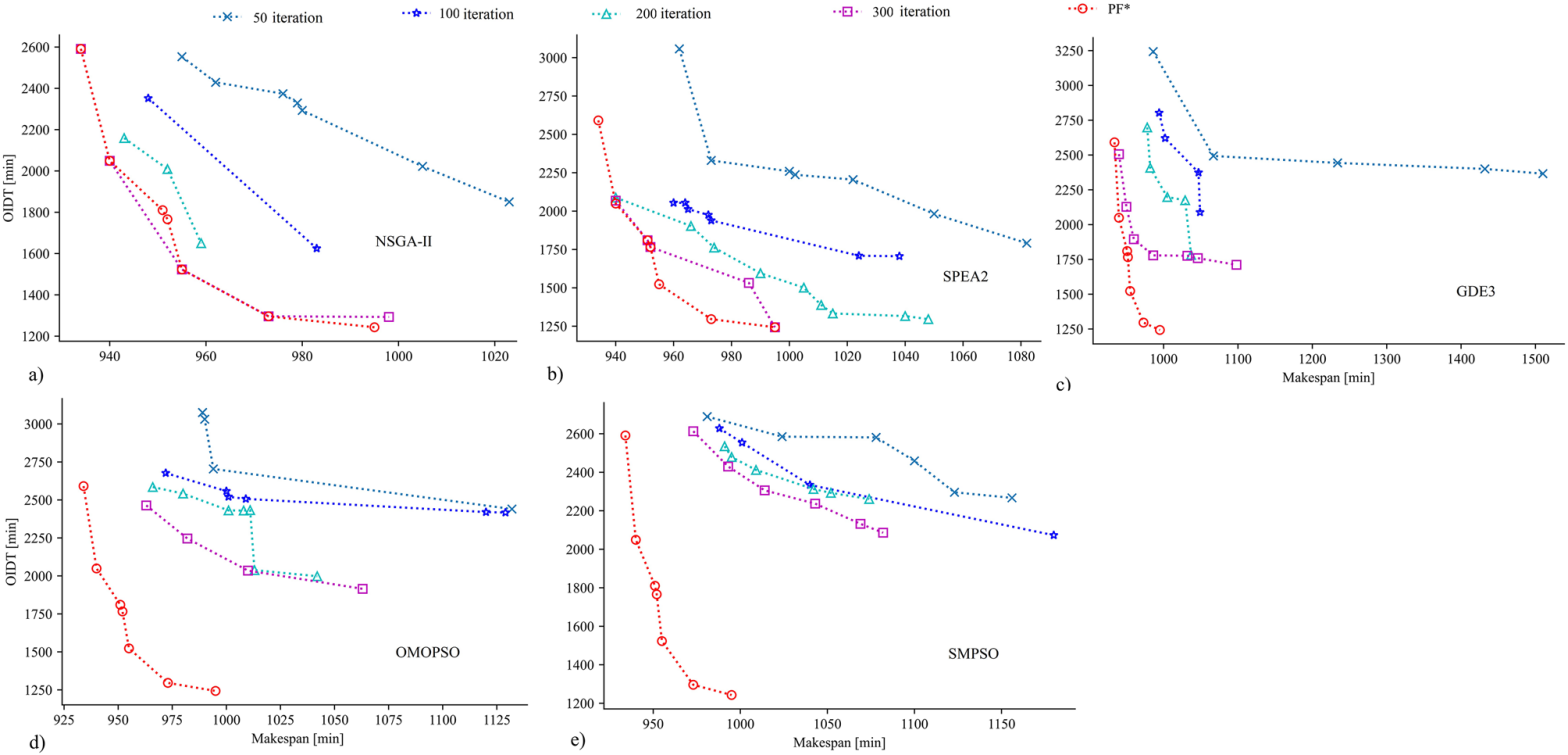
Improvement of candidate Pareto fronts (PF) for BK50 over different iteration size obtained by (**a**) NSGA-II, (**b**) SPEA2, (**c**) GDE3, (**d**) OMOPSO and (**e**) SMPSO. A label of 50 iteration indicates the front is obtained by and algorithm with 50 iterations.

Figure 10 shows the improvement of candidate fronts obtained by algorithms over different iteration sizes for BK50. NSGA-II, SPEA2, and GDE3 improved solution quality remarkably over different iteration sizes (Fig. 10a–c, respectively). However, fronts from OMOPSO and SMPSO were similar, and both displayed poor improvement. All the solutions in PF* for BK50 were obtained by NSGA-II and SPEA2 combinedly, while GDE3 featured a few solutions near PF*. In contrast, no contribution in PF* was observed from MOPSO and SMPSO. Comparable results were obtained for BK15 (not shown). For BK40, GDE3, OMOPSO, and SMPSO obtained Pareto solutions to form PF* and no contribution from NSGA-II and SPEA2 was observed (not shown).

Hecker et al.^6^ used single objective optimization methods to reduce the makespan of BK40, and the results showed that a modified genetic algorithm obtained a minimum makespan of 1261 min 4 times out of 21 runs. Solutions with the best makespan were compared even though multi-objective solutions were found in this study. The best makespan of 1259 min was attained by NSGA-II and SMPSO four times out of four separate runs with varying iteration sizes, while SPEA2, GDE3, and OMOPSO achieved this three times each.

### Pareto domination strength and maximum spread of the solutions

Table 4 shows the pareto domination strength and maximum spread of the solution for the algorithms. According to Pareto domination strength, SPEA2, GDE3, and OMOPSO were observed to perform better for solving BK15, with the worst being SMPSO. For BK40, SPEA2 and GDE3 improved solution quality consistently over different iteration sizes. In contrast, NSGA-II performed worst. The Pareto domination strength of NSGA-II and SPEA2 to solve BK50 was better and found no significant difference between them. Similarly, the difference between the Pareto domination strength of GDE3, OMOPSO, and SMPSO is minor and performed worst. According to this performance metric, only SPEA2 showed better performance in all instances.

**Table 4:** Calculated Pareto domination strength and maximum spread of solutions in front for algorithms.

Problem	Algorithm	Pareto domination strength				Maximum spread of solutions			
		Iteration				Iteration			
		50	100	200	300	50	100	200	300
BK15	NSGA-II	0.33	0.33	0.20	0.00	1.13	1.84	0.17	0.80
	SPEA2	0.33	0.11	0.17	− 0.33	0.97	0.23	0.37	1.00
	GDE3	0.22	0.25	0.33	0.00	0.15	0.13	0.16	0.46
	OMOPSO	0.24	0.17	0.00	0.00	0.10	0.08	0.20	0.51
	SMPSO	0.33	0.33	0.33	0.33	0.44	0.29	0.28	0.06
BK40	NSGA-II	0.17	0.22	0.11	0.11	0.81	0.25	0.44	0.71
	SPEA2	− 0.33	− 0.33	− 0.33	− 0.33	0.00	0.00	0.00	0.00
	GDE3	− 0.33	− 0.11	− 0.33	− 0.33	0.00	0.61	0.61	0.83
	OMOPSO	0.20	0.14	0.17	0.00	0.26	0.66	0.72	0.42
	SMPSO	0.17	− 0.11	0.00	− 0.33	0.32	0.76	0.96	1.00
BK50	NSGA-II	0.10	0.14	0.05	− 0.09	0.82	1.00	1.00	0.98
	SPEA2	0.10	0.10	0.08	− 0.09	0.49	0.78	0.79	1.00
	GDE3	0.14	0.14	0.14	0.10	0.18	0.50	0.66	0.75
	OMOPSO	0.14	0.14	0.14	0.14	0.17	0.48	0.76	0.74
	SMPSO	0.14	0.14	0.10	0.10	0.55	0.66	0.71	0.69

The maximum spread of the solutions in front measures the distribution and spread of candidate solutions over the PF offered by an algorithm, with a greater number indicating better performance. NSGA-II had higher maximum spread of the solutions in all instances and was found to outperform all other algorithms in this performance metric. The maximum spread of the solutions of GDE3 was equivalent to NSGA-II in most circumstances in terms of problems and iteration sizes. But it had the lowest value for BK40 where only one solution was obtained every time. In contrast, GDE3 had better maximum spread of the solutions for BK40. In most scenarios, GDE3 and OMOPSO was remarkably comparable to each other. With the increasing iteration size to solve BK15, the maximum spread of the solutions of SMPSO decreased. It means that with a short iteration size, it was able to find solutions that had better distribution, but they were mostly suboptimal. In contrast, a large iteration size obtained comparatively better solutions, however, their dispersion was poor. For BK40 and BK50, SMPSO had a modest maximum spread of the solutions.

### Distance to Pareto front

**Figure 11:**
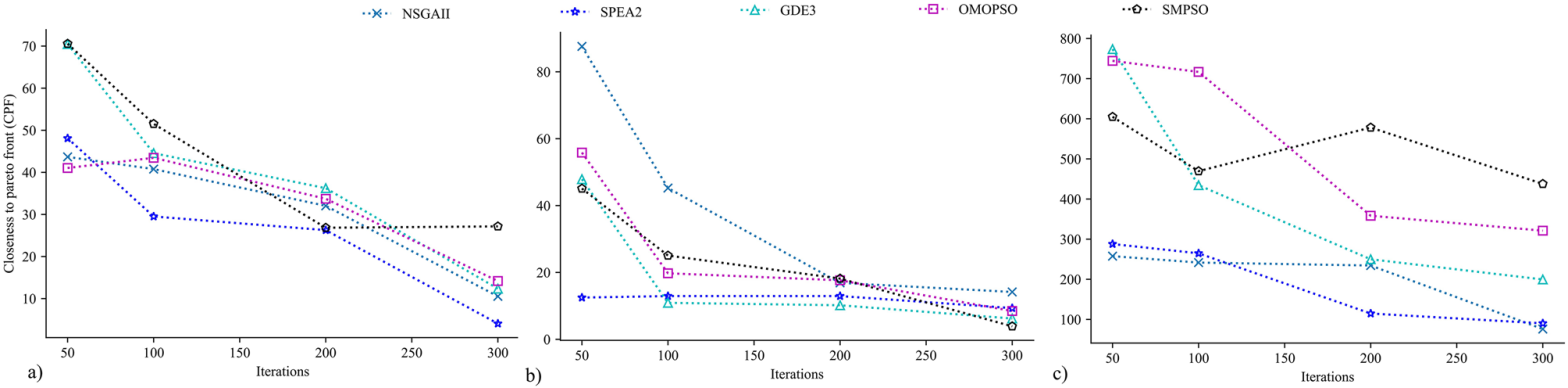
Distance to Pareto front of candidate fronts obtained by algorithms for (**a**) BK15, (**b**) BK40 and (**c**) BK50.

The distance to Pareto front for the algorithms is shown in Fig. 11. This performance metric represents how close a candidate's front to PF* is, where a low value indicates better performance. For BK15, SPEA2 exhibited promising improvement over different iteration sizes, while SMPSO was observed to perform worst (Fig. 11a). There is no substantial difference between the distance to Pareto front of NSGA-II, GDE3, and OMOPSO. For BK40, the PF obtained by SPEA2 had the lowest value with minimum iteration (Fig. 11b). However, with increasing iteration sizes, the distance to Pareto front of GDE3, OMOPSO, and SMPSO was comparable to that of SPEA2. According to this performance indicator, NSGA-II had the worst distance to Pareto front with 50 iterations, which sharply improved with increased iteration sizes, yet could not outperform any algorithms. Figure 11c shows the distance to Pareto front of algorithms for BK50, where NSGA-II and SPEA2 outperformed GDE3, OMOPSO, and SMPSO. The values of BK50 distinguished algorithms’ performance at every iteration which was not prominent for BK15 (Fig. 11a) and BK40 (Fig. 11b).

### Relative hyper area

**Figure 12:**
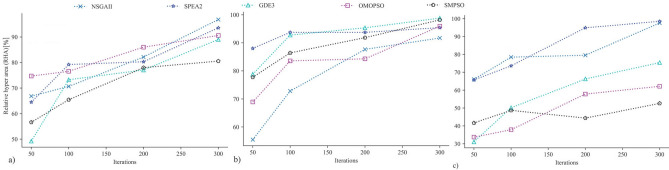
Relative hyper area (RHA) of candidate Pareto fronts (PF) obtained by algorithms for (**a**) BK15, (**b**) BK40 and (**c**) BK50.

Figure 12 presents the relative hyper area of algorithms. It measures convergence and distribution of algorithms with a higher value indicating better performance. NSGA-II had higher value for BK15 and BK50, while for BK40 it performed worst, and SPEA2 had higher relative hyper area for all the cases. GDE3 and OMOPSO had moderate relative hyper area for all instances. In contrast, SMPSO showed the lowest relative hyper area for BK15 and BK50, and higher for BK40.

The distance to Pareto front and the relative hyper area for BK15 and BK40 illustrate the significant improvement in solutions’ quality over different iteration sizes for algorithms (Figs. 11, 12). With the higher iteration size, the performance difference between algorithms was found to be minimum. Only NSGA-II, SPEA2, and GDE3 were able to follow this trend in BK50, while OMOPSO and SMPSO fell behind. One reason could be that there are more local minima in the solution space of BK50 compared to that of BK15 and BK40. Many suboptimal solutions exist for BK50 with higher OIDT with a small difference in makespan (Fig. 8c). Additionally, BK50 has a higher dimension—maximum product groups—to optimize.

### Performance evaluation of algorithms

**Figure 13:**
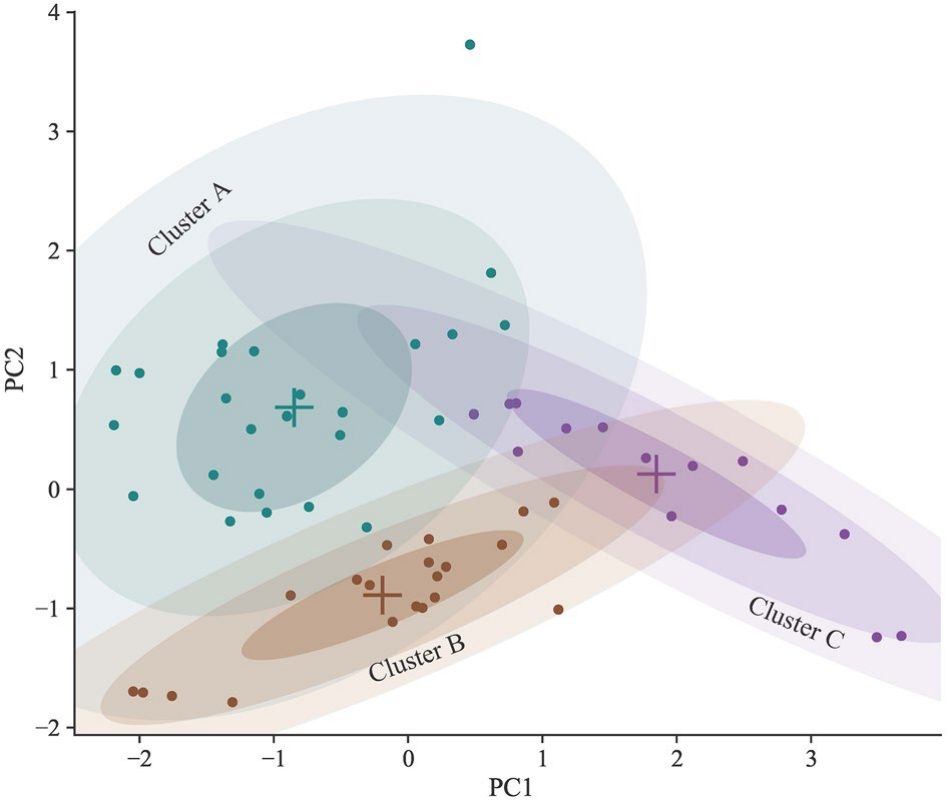
Clusters of algorithms’ solutions found by Gaussian mixture model.

Performance metrics explain a specific feature of solution quality. The solutions, however, can be categorized into different quality levels using a clustering approach. The frequency with which an algorithm produces good or poor-quality solutions is a measure of its efficiency. Initially, the performance metrics for all instances are used to perform principal component analysis (PCA)^54^. Two principal components (PC1 and PC2) with higher variances were taken to perform a Gaussian mixture model for clustering^22^. Figure 13 shows three clusters. The clusters' solutions were identified using the associated labels, which refer to instances, algorithms, and iteration sizes such as BK15, NSGA-II, and 50, respectively. The quality of different clusters were determined based on corresponding performance metrics where Cluster A represents better performances, and Cluster B and Cluster C show moderate and worst performances, respectively.

**Figure 14:**
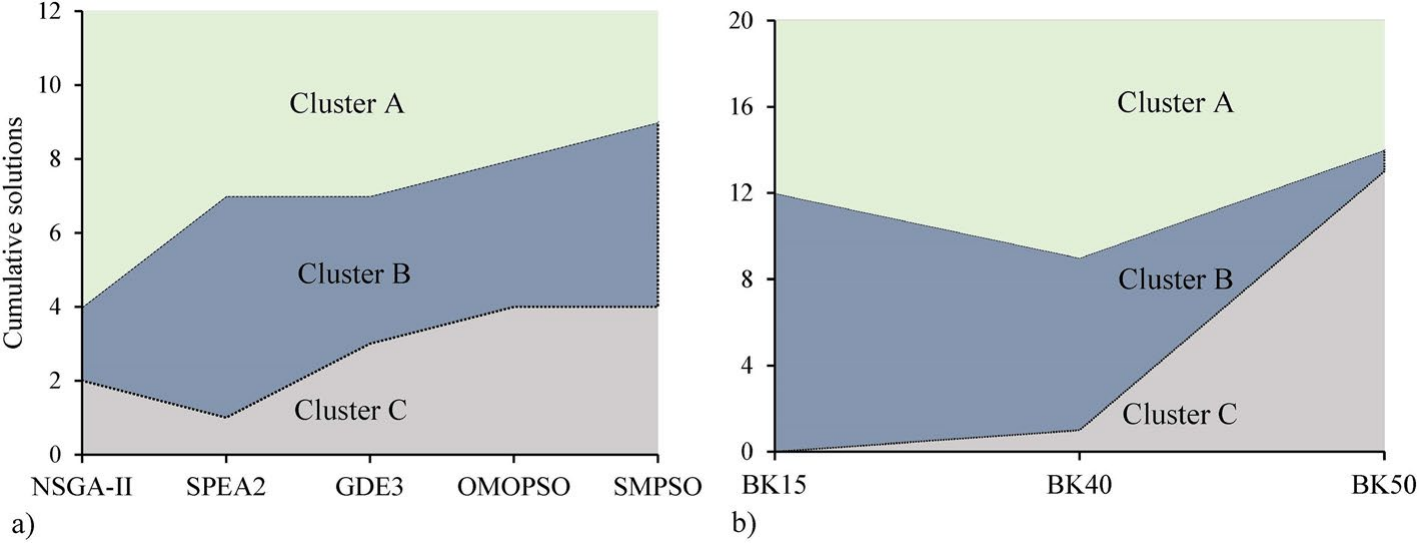
Distribution of obtained solutions among clusters with respect to: (**a**) algorithms (**b**) problems.

Figure 14 represents the distribution of obtained solutions into three clusters. Cluster A has 25 high-quality solutions, Cluster B has 21, and Cluster C has 14 low-quality solutions for the instances. NSGA-II had the highest number of solutions in Cluster A, followed by SPEA2 and GDE3 (Fig. 14a). In contrast, OMOPSO and SMPSO have the lowest number of solutions in this cluster. NSGA-II, with only four moderate and worst solutions, outperformed all other algorithms. SPEA2, with the highest number of moderate and lowest worst solutions, followed NSGA-II. In terms of distribution of obtained solutions among clusters, GDE3 performed slightly better than OMOPSO. In comparison to NSGA-II and SPEA2, SMPSO has the lowest solutions in Cluster A and the highest in Cluster C, indicating worse performance. For BK15, a large number of obtained solutions were moderate, with no worst solution (Fig. 14b). In contrast, for BK40, the majority of solutions fell into cluster A, emphasizing a problem that is comparatively easy to solve. BK50 revealed a considerable rise in the difficulty of obtaining moderate and better solutions, with Cluster C accounting for 65% of all solutions. Three of the six solutions in Cluster A were achieved by NSGA-II, two by SPEA2, and one by GDE3. In Cluster B, there is only one solution for BK50, which was obtained by SPEA2. In contrast, all the solutions from OMOPSO and SMPSO are in Cluster C. With only one Cluster C solution for BK50, NSGA-II and SPEA2 displayed consistently better performance. According to the cluster analysis, NSGA-II outperformed all other algorithms, followed by SPEA2. GDE3 performed better than OMOPSO and SMPSO, but OMOPSO and SMPSO showed no notable difference in performance.

The comparison of computation time of algorithms is performed with 50 iterations for the instances. Although OMOPSO needed the shortest calculation time (12 min, 58 min, 324 min for BK15, BK40 and BK50, respectively), the difference between it and other methods is insignificant. It took roughly the same amount of time for NSGA-II and SPEA2 in each case—13 min, 62 min, and 342 min, respectively. GDE3 showed slightly lower computation time with instances taking 12 min, 61 min, and 337 min, respectively. SMPSO, in contrast, had the longest computing time for every instance (13 min, 67 min, and 360 min, respectively). In comparison to OMOPSO, the extension to SMPSO appears to have triggered slightly high computing time as velocity constraints are applied to each iteration and dimension of the problem.

The current study shows that production planning using a flow shop model is feasible in practice when considering the actual resource limitations in bakeries. Along with makespan, minimizing the oven idle time also offers the potential to substantially lower manufacturing costs. To improve the current state of production efficiency in real cases from bakeries, multi-objective optimization algorithms were integrated with hybrid no-wait flow shop model. Among them, NSGA-II performed better in solving problems of various dimensions. Moreover, when multiple products share a predecessor, the increased oven idle time results in energy loss. Therefore, wherever possible, it is suggested to keep the processing route for a product separate from other products. Six bakery production datasets from Denmark were used by Babor et al.^55^ to increase the production efficiency. The results revealed that NSGA-II performed efficiently to reduce makespan by up to 12% and oven idle time by up to 61%. Particle swarm optimization was used in a study^5^ to obtain the best planning for a bakery’s production in Spain. The optimum solution, according to the results, minimized the makespan by 29% and the oven idle time by 8%.

## Conclusions and future works

In this paper, three production optimization problems from small and medium-sized bakeries were investigated. The objectives of optimization were to minimize simultaneously makespan and oven idle time (OIDT). A hybrid no-wait flow shop scheduling model with all constraints encountered in practice was implemented to simulate the bakery schedule. The optimum schedules were found using five multi-objective optimization algorithms: non-dominated sorting genetic algorithm (NSGA-II), strength Pareto evolutionary algorithm (SPEA2), generalized differential evolution (GDE3), improved multi-objective particle swarm optimization (OMOPSO), and speed-constrained multi-objective particle swarm optimization (SMPSO). To compare the efficiency of the algorithms, each problem was solved with different iteration sizes.

The computational results revealed that the shape of a true Pareto front is determined by the characteristics of the problems, such as the number of items, product interdependency, and alternative machinery. Although makespan has the most influence on production expenditure, it was observed that a substantial reduction in OIDT is possible. Many solutions with the shortest makespan had higher OIDT (up to 231 min) that showed significant energy waste and CO_2_ emissions. Therefore, with the same makespan, multi-objective algorithms can provide solutions with reduced energy waste. Furthermore, many Pareto solutions, aside from the one with the shortest makespan, provide better tradeoffs between makespan and OIDT. It means that by losing a very marginal amount in makespan, some solutions offer a substantial reduction in OIDT. BK50 showed an additional 1348 min of oven idle time can be reduced if the makespan is increased by 61 min. Therefore, the overall production expenditure can be significantly minimized. Product group formulation may influence OIDT. In the best-case scenario, for BK40 with no predecessor in any group, a schedule with 0 min OIDT is possible. However, because many products have a few combined initial processing stages, for BK50 the lowest possible OIDT is 1243 min, resulting in significant energy loss.

NSGA-II outperformed other algorithms by obtaining a smaller number of poor solutions and a high number of better solutions. SPEA2 followed NSGA-II by delivering promising solutions. GDE3 performed slightly better than OMOPSO and SMPSO. The performance of OMOPSO and SMPSO was poor to solve the instances and no significant difference between them was observed. However, OMOPSO had the lowest computation time while SMPSO had approximately 11% higher computation time due to the addition of velocity constraints.

The deterministic duration of the processing tasks and the absence of machine maintenance or failure assumed in this study may not reflect many realistic production problems. Based on prior relevant studies^56,57^, the effects of non-deterministic processing duration and machine disturbances on the production efficiency of bakeries could be an interesting subject for future research.

## Figures and Tables

**Figure 15:**
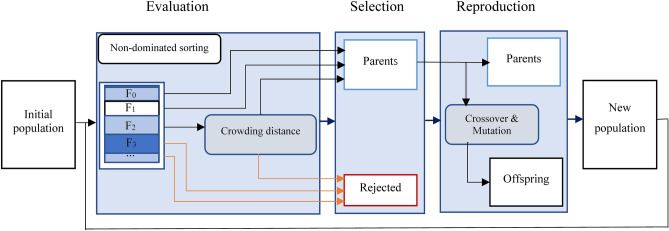
An evaluation procedure of the NSGA-II^55^. Non-dominated sorting divides the population into different ranks (F_0_, F_1_, F_2_, …). Individuals from the same optimal front are maintained in one rank.

**Figure 16:**
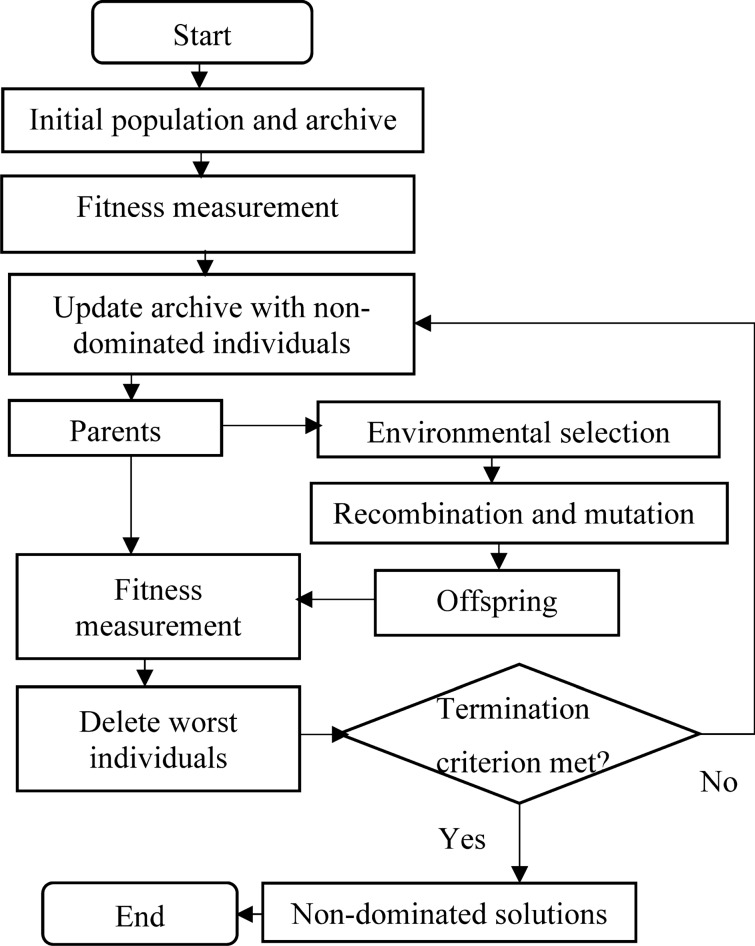
Flowchart of strength Pareto evolutionary algorithm (SPEA2).

**Figure 17:**
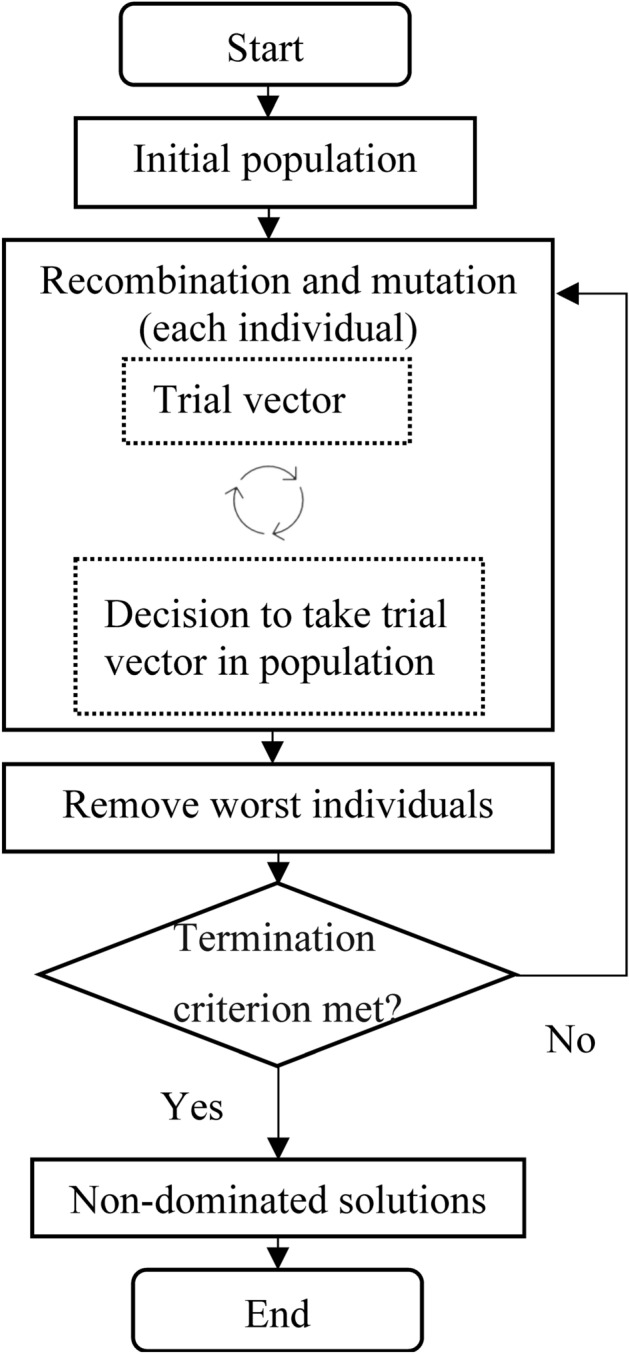
Optimization procedure for generalized differential evolution (GDE3).

**Figure 18:**
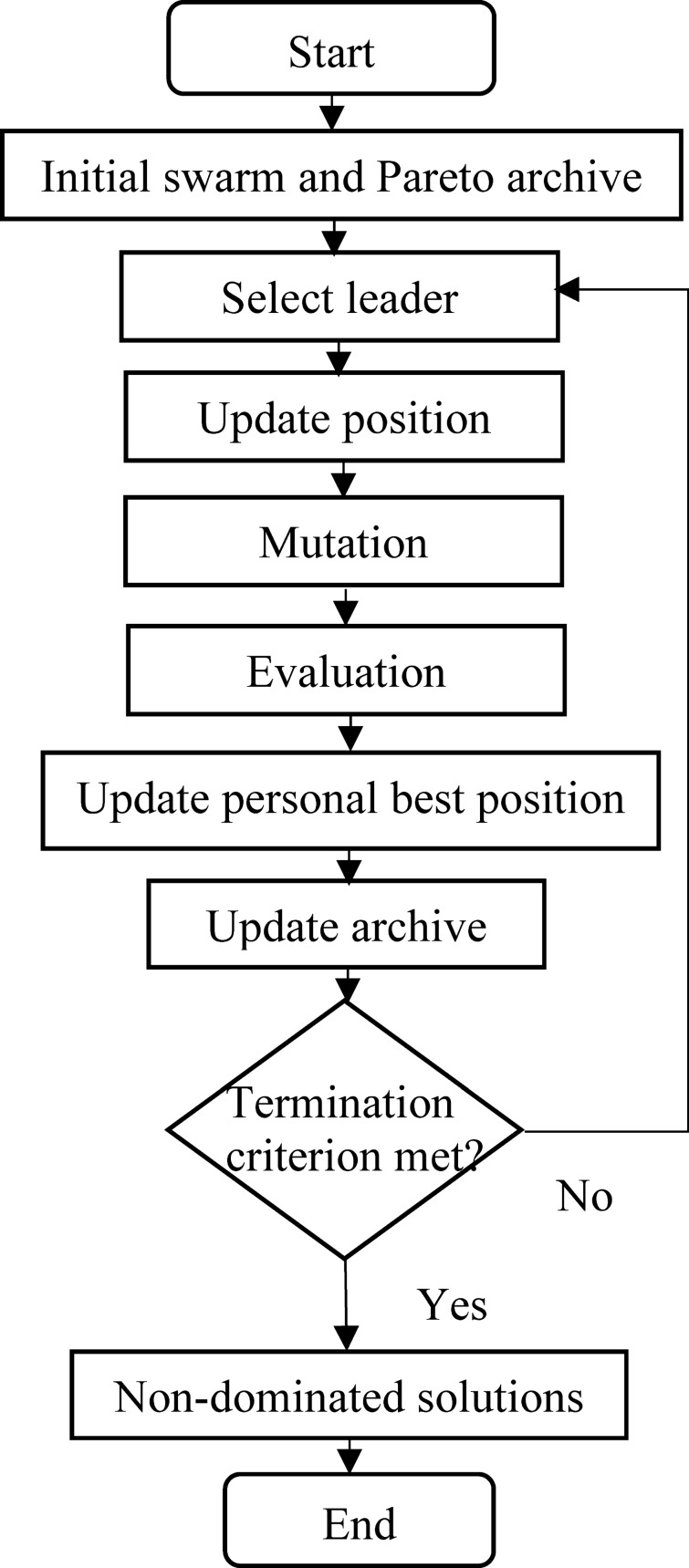
Flowchart of improved multi-objective particle swarm optimization algorithm (OMOPSO) to obtain non-dominated solutions.

## Data Availability

For this study, production data from bakeries in Europe were used. BK40 was collected and analyzed by Hecker et al.^6^, whereas BK15 and BK50 are publicly accessible^25^. The production data are available from the corresponding author on reasonable request.

## Multi-objective optimization algorithms

### Non-dominated sorting genetic algorithm (NSGA-II)

The following is the description of the NSGA-II (Fig. 15) proposed by Deb et al.^27^.

Create a random population of size $NP$. Each individual in the population is a candidate solution vector to a problem. Assess the individuals in the population using HNFSM by calculating objective values.

1. The objective values are used to build fitness vectors. The population is sorted into distinct rankings using the fast non-dominated sorting strategy. Using the Pareto dominance operator, each individual's fitness is compared to that of others. The rank of an individual is determined by the number of other individuals who dominate it, which is known as the sum of domination. If the sum of domination for an individual is 0, it is called a non-dominated solution or Pareto optimal solution. Individuals with a higher sum of domination have a suboptimal solution to the problem.
2. A binary tournament selection process is used to choose two parents from the existing population for creating two offspring. In the binary tournament selection process, four individuals from the current population are picked and the one with the best rank is chosen as one of the parents. The same procedure is followed to complete the parents’ poll to perform crossover and mutation. It is repeated to create new offspring of *NP* size.
3. Evaluate offspring to get the corresponding fitness vectors. The offspring and parent population are combined. During this phase, the population doubles in size.
4. The combined population is sorted into different ranks using the fast non-dominated sorting approach.
5. To select the best population of $NP$ size, the individuals with the best rank are chosen first. If the best rank does not have enough individuals to fill all the empty slots of the best population, the individuals of subsequent ranks are chosen. If a rank has more individuals than empty slots, the crowding distance operator is used to select individuals from the less crowded part of the objective space. The crowding distance is set to infinity for border solutions of a rank to give preference over others. All the solutions of the rank are sorted in descending order of crowding distance. Individuals with a higher crowding distance fill the empty slot first until the best population size reaches $n$.
6. Employ crossover and mutation operators to produce $NP$ offspring from the best population.
7. Repeat steps 4–7 until the termination criterion is met.

### A.2: Strength Pareto evolutionary algorithm (SPEA2)

The process flowchart for SPEA2 is shown in Fig. 16. The brief of SPEA2^32^ is as follows:

1. Generate an initial population of size $N$ where each individual is a candidate solution vector for a problem. Create an empty external archive. Set the size limit for external archives to $EA$. In this archive, the best individuals are stored.
2. Evaluate the individuals to get fitness values. The fitness of the individuals in the population is calculated. Initially, a strength value for each individual is calculated using Eq. (A1).
  A1 $$ S\left( i \right) = \left| { \left\{ {j | j \in PO_{G} + AP_{G} \wedge i \succ j} \right\} } \right| $$
  where $\left|.\right|$ indicates the cardinality of a set, $+$ sign is for multiset union, $\succ$ symbolizes the Pareto dominance relation, ${PO}_{G}$ is the population and ${AP}_{G}$ is archive size at generation $G$. Therefore, $S\left(i\right)$ represents the number of other individuals $(j)$ in the population and archive that are dominated by an individual $i$.

The raw fitness of individual $i$ is defined by Eq. (A2).

A2 $$ R\left( i \right) = \sum\nolimits_{{j \in PO_{G} + AP_{G} , j \succ i}} {S\left( j \right)} $$

Equation (3) implies that the raw fitness of an individual is the sum of the strength of its dominators. In the next stage, a density estimation approach is employed. For that purpose, *k*th nearest neighbor method is adapted. The distances between $i$ and all other individuals $\left(j\right)$ in objective space are calculated. The distance list is sorted in increasing order. The *k*th element gives the distance sought ${\sigma }_{i}^{k}$. Equation (A3) defines the density calculation for an individual.

A3 $$ D\left( i \right) = \frac{1}{{\sigma_{i}^{k} + 2}} $$

where $k= \sqrt{NP+ EA}$, $NP$ and $EA$ are the size of the total population and archive, respectively.

Finally, the fitness of an individual $i$ can be stated as follows:

A4 $$ F\left( i \right) = R\left( i \right) + D\left( i \right) $$

3. Take all non-dominated individuals to archive. If the size of the archive exceeds the limit, reduce it by using a truncation operator that prevents boundary solutions from being removed. If the size is less than the limit, fill it with dominated individuals.
4. Employ binary tournament selection with replacement to obtain offspring. Initially, all individuals are compared based on Pareto dominance. Rank the individuals depending on their domination level. Afterward, estimate their density information within the corresponding rank. It represents the sum of distances between the two closest individuals along with each objective. Based on these two sorting approaches, the parent selection is performed. Apply recombination and mutation operators to keep high diversity among the population.
5. Combine offspring, parent population, and archived individuals. Delete the worst 50% of combined population based on their fitness values (as shown in step 2).
6. Continue with steps 3–5 until a stopping criterion is met.

### A.3: Generalized differential evolution (GDE3)

The differential evolution algorithm was first introduced by Storn and Price^58^. Like all other evolutionary algorithms, it has a random initial population, which is improved over the generations. It features cross-over, mutation, and selection operators to improve the solution. The selection rule is one of the key differences compared to other evolutionary algorithms. It is the process to decide whether a new individual should replace one from the population to generate efficient individuals for the next generation. The decision is taken based on some constraints that are regulated by crossover constant and differential variation between two individuals. Later, differential evolution algorithm was extended to generalized differential evolution (GDE) for multi-objective optimization problems by modifying the selection rule^59^. The optimization procedure of GDE3 is presented in Fig. 17. GDE3, an improved version, can be described as follows^36^.

1. Initialize population of size *NP* ($NP\ge 4)$*,* amplification constant for differential variation, $F\in \left(0, 1+\right]$, crossover constant, $CR \in \left[0, 1\right]$, dimensions or parameters of the problem, $D$, the maximum generation ${G}_{max}$, and a constant $A= 0$. The solution vector is ${\overrightarrow{x}}_{i,G}$ for an individual $i$ at $G$ generation where $i=\left\{1, 2, \dots , NP\right\}$, and $G=\left\{1, 2, \dots ,{G}_{max}\right\}$. The value of $d$ dimension in $i$ individual at $G$ generation is indicated by ${x}_{d,i,G}$, where $d=\left\{1, 2, \dots , D\right\}$.
2. Mutate and recombine each individual in the population. For an individual $i$, chose three different individuals randomly ${R}_{1}, {R}_{2},{R}_{3}$ where ${R}_{1}\ne {R}_{2}\ne {R}_{3}\ne i$ and ${R}_{1}, {R}_{2},{R}_{3}, i\in \left\{1, 2, \dots , NP\right\}$. Chose random parameter ${d}_{rand}$ where ${d}_{rand}\in \left\{1, 2,\dots , D\right\}$. For each dimension $\left(d\right)$, the following procedure is applied to ${\overrightarrow{x}}_{i,G}$ to get a new individual, which is known as a trial vector $\left({\overrightarrow{u}}_{i,G}\right)$.
  A5 $$ u_{{d,{ }i,G}} = { }\left\{ {\begin{array}{*{20}l} {x_{{d,{ }R_{3,} G}} + F \times \left( {x_{{d,{ }R_{1,} G}} - { }x_{{d,{ }R_{2,} G}} } \right)} \hfill & {if r < CR or d = d_{rand} } \hfill \\ {x_{{d,{ }i,G}} } \hfill & {otherwise} \hfill \\ \end{array} } \right. $$
  where $r$ is a random value between 0 and 1 and ${d}_{rand}\in \left\{1, 2, .., D\right\}$
3. Decide whether the trial vector should become a member of generation $G+1$.
  A6 $$ \vec{x}_{i,G + 1} = \left\{ {\begin{array}{*{20}l} {\vec{u}_{i,G} } \hfill & { if \overrightarrow { u}_{i,G} \underline { \prec }_{c} \vec{x}_{ i,G} } \hfill \\ {\vec{x}_{ i,G} } \hfill & {otherwise} \hfill \\ \end{array} } \right. $$
  where the symbol ${\preccurlyeq }_{c}$ is constraint domination. To define it, ${\overrightarrow{u}}_{i,G}$ constraint dominates ${\overrightarrow{x}}_{ i,G}$ if any of the following conditions is true^60^:

${\overrightarrow{u}}_{i,G}$ is feasible and ${\overrightarrow{x}}_{ i,G}$ is not.

${\overrightarrow{u}}_{i,G}$ and ${\overrightarrow{x}}_{ i,G}$ are infeasible and ${\overrightarrow{u}}_{i,G}$ dominates ${\overrightarrow{x}}_{ i,G}$ in constraint function space.

${\overrightarrow{u}}_{i,G}$ and ${\overrightarrow{x}}_{ i,G}$ are feasible and ${\overrightarrow{u}}_{i,G}$ dominates ${\overrightarrow{x}}_{ i,G}$ in objective space.

Set the following conditions:

A7

where ${C}_{d}\left({\overrightarrow{u}}_{i,G}\right)$ indicates constraint associated with $d$th dimension.

4. Repeat steps 2–3 $NP$ times to complete mutation and recombination of the population of the generation $G$.
5. Select individuals $\left(\overrightarrow{x}\right)$ that meet the following condition:
  A8
  where $CD$ is crowding distance that measures the crowdedness of a vector in a non-dominated set.
6. Remove the individuals in $\overrightarrow{x}$ from population. Set $A=A-1$ and repeat step 5 while $A>0$.
7. Increase the generation from $G$ to $G+1$.
8. Repeat the steps 2 − 7 while $G\le {G}_{max}$.

### A.4: Improved multi-objective particle swarm optimization (OMOPSO)

Figure 18 shows the flowchart for OMOPSO. The following is the description of OMOPSO proposed by Sierra and Coello^44^.

1. Initialize a swarm where each particle in the swarm is a candidate solution vector to solve a problem. Evaluate them and initialize the best position and velocity of the particles. Set the size for leaders and initialize leaders from the existing swarm. Save the leaders in the Pareto archive. Initialize generations, $G=\left\{1, 2, \dots , {G}_{max}\right\}$. The crowdedness of the leaders is calculated.
2. For each particle select a leader through a binary tournament. The selection criterion is the crowding distances where a leader with a higher crowding distance is chosen.
3. Update the position of the particle to a new position using Eqs. (A9), (A10).
  A9 $$ \vec{v}_{i,G} = W \times \vec{v}_{i,G - 1} + { }C_{1} \times r_{1} \times \left( {\vec{x}_{i,best} - \vec{x}_{i,G - 1} } \right) + C_{2} \times r_{1} \times \left( {\vec{L}_{h} - \vec{x}_{i,G - 1} } \right) $$
  A10 $$ \vec{x}_{i,G} = \vec{x}_{i,G - 1} + \vec{v}_{i,G} $$
  where $i$ indicates one particle, $\overrightarrow{v}$ is the velocity, $W$ is the inertia weight, ${C}_{1}$ and ${C}_{2}$ are velocity control parameters, ${r}_{1}$ and ${r}_{2}$ are random numbers between 0 and 1, ${\overrightarrow{x}}_{i,best}$ and ${\overrightarrow{x}}_{i,G}$ are particle’s best position and current position at generation $G$, respectively, ${\overrightarrow{L}}_{h}$ is a position vector of the selected leader of the $h$ index from the Pareto archive.
4. Divide swarm into three parts to employ distinct mutation treatments: no mutation, uniform mutation, and non-uniform mutation.
5. The particles are evaluated. The personal best position $\left({x}_{best}\right)$ for each particle is updated by comparing the current and personal best fitness.
6. Update leader set in archive by including non-dominated Pareto solutions and removing dominated solutions. Calculate the crowding distance of the leaders. Eliminate leaders based on crowding distance if the size of the archive exceeds the limit. Increase the generation from $G$ to $G+1$.
7. Repeat the process (steps 2–6) until $G$ reaches ${G}_{max}$. Save the Pareto archive as the set of optimal solutions to the problem.

### A.5: Speed-constrained multi-objective particle swarm optimization (SMPSO)

SMPSO, an extended version of OMOPSO, was proposed by Nebro et al.^42^. In this proposal, the values for velocity parameters in Eq. (10), ${C}_{1}$ and ${C}_{2}$ are controlled by using following constriction coefficient $(\chi )$ calculated by Eq. (A11).

A11 $$ \chi = { }\frac{2}{{2 - { }\varphi - { }\sqrt {\varphi^{2} - 4\varphi } }} $$

where $$\varphi = \left\{ {\begin{array}{*{20}l} {C_{1} + C_{2} } \hfill & {if C_{1} + C_{2} > 4} \hfill \\ 1 \hfill & {otherwise} \hfill \\ \end{array} } \right.$$

The velocity of the particle in each parameter $d$ is bounded using the following velocity constriction equations (Eqs. A12, A13):

A12 $$ v_{i,d,G} = \left\{ {\begin{array}{*{20}l} {delta_{d} } \hfill & {if v_{i,d,G} > delta_{d} } \hfill \\ { - delta_{d} } \hfill & {if v_{i,d,G} \le delta_{d} } \hfill \\ {v_{i,d,G} } \hfill & {otherwise} \hfill \\ \end{array} } \right. $$

A13 $$ delta_{d} = \frac{{UB_{d} - LB_{d} }}{2} $$

where ${UB}_{d}$, and ${LB}_{d}$ are upper bound and lower bound of the parameter $d$.

To summarize the modifications in SMPSO from OMOPSO, for each particle, the velocity is calculated by Eq. (A10), which is then multiplied by the constriction coefficient $(\chi )$ (Eq. A11). The resulting value for each parameter is constrained by Eqs. (A12), (A13). The rest of the procedure is the same as OMOPSO. Table 5 shows the parameters setting of the algorithms.

**Table 5:** Parameters setting for the algorithms.

Parameters	NSGA-II, SPEA2	GDE3	OMOPSO	SMPSO
Population size	50	50	50	50
Mutations	Polynomial	–	Uniform, non-uniform	Polynomial mutation
Mutation rate	$\frac{1}{\mathrm{Product groups}}$	–	$\frac{1}{\mathrm{Product groups}}$	$\frac{1}{\mathrm{Product groups}}$
Crossover	Simulated binary	Differential evolution	–	–
Crossover rate	1	1	–	–
Selection	Binary tournament	Differential evolution	–	–
Other parameters		CR = 1 F = 0.4	C1 = random (1.5, 2) C2 = random (1.5, 2) W = random (0.1, 0.5)	C1 = random (1, 2.5) C2 = random (1, 2.5) W = 0.2
